# Midbrain injury in patients with subarachnoid hemorrhage: a diffusion tensor imaging study

**DOI:** 10.1038/s41598-021-03747-1

**Published:** 2022-01-07

**Authors:** Sung Ho Jang, Young Hyeon Kwon

**Affiliations:** Department of Physical Medicine and Rehabilitation, College of Medicine, Yeungnam University, 317-1, Daemyungdong, Namku, Daegu, 705-717 Republic of Korea

**Keywords:** Neuroscience, Neurology

## Abstract

We investigated the characteristics of midbrain injuries in patients with spontaneous subarachnoid hemorrhage (SAH) by using diffusion tensor imaging (DTI). Twenty-seven patients with SAH and 25 healthy control subjects were recruited for this study. Fractional anisotropy (FA) and mean diffusivity (MD) data were obtained for four regions of the midbrain (the anterior ventral midbrain, posterior ventral midbrain, tegmentum area, and tectum) in 27 hemispheres that did not show any pathology other than SAH. The mean FA and MD values of the four regions of the midbrain (anterior ventral midbrain, posterior ventral midbrain, tegmentum, and tectum) of the patient group were significantly lower and higher than those of the control group, respectively (*p* < 0.05). The mean FA values of the patient group were significantly different among the anterior ventral midbrain, posterior ventral midbrain, tegmentum, and tectum regions (ANOVA; F = 3.22, *p* < 0.05). Post hoc testing showed that the mean FA value of the anterior ventral midbrain was significantly lower than those of the posterior ventral midbrain, tegmentum, and tectum (*p* < 0.05); in contrast, there were no differences in mean FA values of the posterior ventral midbrain, tegmentum, and tectum (*p* > 0.05). However, differences were not observed among four regions of the midbrain (anterior ventral midbrain, posterior ventral midbrain, tegmentum, and tectum) in the mean MD values. We detected evidence of neural injury in all four regions of the midbrain of patients with SAH, and the anterior ventral midbrain was the most severely injured among four regions of the midbrain. Our results suggest that a pathophysiological mechanism of these neural injuries might be related to the occurrence of a subarachnoid hematoma.

## Introduction

Spontaneous subarachnoid hemorrhage (SAH), which appears in 5% of all stroke patients, is the extravasation of blood into the subarachnoid space between the arachnoid membrane and the pia mater in the brain, mainly occurs following rupture of an aneurysm^1,2^. More than 50% of patients with SAH are reported to present with various neurological complications, which suggests the presence of SAH-related brain injuries such as cognitive impairment, motor weakness, consciousness impairment, visual problem, and somatosensory deficit^3–16^. Previous studies have demonstrated the presence of SAH-related brain injury by using functional neuroimaging methods such as functional magnetic resonance imaging (MRI), positron emission tomography, or single-photon emission computed tomography^17–20^. However, these imaging methods are limited in their ability to clearly discriminate a specific neural structure from adjacent neural structures.

By contrast, diffusion tensor imaging (DTI) has a unique advantage in its ability to identify microstructural white matter abnormalities that are not usually detectable on conventional brain MRI^21–24^. DTI allows evaluation of the integrity of the white matter of the brain by virtue of its ability to image water diffusion characteristics by assessing the appropriated DTI parameters^21–24^. Many studies have demonstrated SAH-related brain injury based on DTI results^5–16^. Although the precise pathophysiological mechanisms of SAH-related brain injury have not been clearly clarified, several DTI-based studies have demonstrated that the neural structures that are located adjacent to the subarachnoid space can be injured by a subarachnoid hematoma^7–15^. Regarding the midbrain, which is interfaced with the perimesencephalic cistern (a large subarachnoid cistern), a few studies have used DTI to demonstrate injuries of neural tracts that pass through the midbrain^7,9,10,25,26^. However, no study on the effect of SAH on the whole midbrain has been reported. We hypothesized that the neural areas that are close to the subarachnoid space would be more vulnerable than neural areas that are far from the subarachnoid space and integrity of the white matter would be affected according to the distance from the subarachnoid space.

In the current study, by using DTI, we investigated the characteristics of midbrain injuries in patients with SAH.

## Methods

### Subjects

A total of 27 consecutive patients (10 men, 17 women; mean age 55.44 ± 9.75 years; range, 35–77 years) with spontaneous SAH prior to the time of DTI scanning and 25 age and sex-matched healthy control subjects (13 men, 12 women; mean age, 52.16 ± 9.96 years; range, 40 – 77) with no history of neurologic/psychiatric disease were recruited. Inclusion criteria for the 27 patients were as follow: (1) first-ever stroke; (2) SAH or SAH with spontaneous intracerebral hemorrhage confined to a unilateral supratentorial and infratentorial areas which was confirmed by a neuroradiologist; (3) DTI scans obtained during the chronic stage of SAH and more than 2 weeks after SAH onset; (4) age at the time of SAH: 20–79 years; and (5) no previous history of neurologic/psychiatric disease. Demographic data of the patient and control groups are summarized in Table 1. No significant differences in age, sex, or hemisphere ratio were detected between the patient and control groups (*p* > 0.05). This study was performed retrospectively and conducted in accordance with the recommendations of the institutional review board of Yeungnam University Hospital. All of the patients and control subjects provided signed, informed consent and the institutional review board of Yeungnam University hospital approved the study protocol (ethical approval number: YUMC-2021-03-014).

**Table 1:** Demographic data for the patient and control groups.

	Patient group (*n* = 27)	Control group (*n* = 25)
Age (years)	55.44 ± 9.75	52.16 ± 9.96
Sex, male/female	10 / 17	13/12
Duration from SAH onset (months)	2.18 ± 2.12	
Modified Fisher grade of SAH	2.70 ± 1.20	
Ruptured artery (ACoA:ACA:MCA:PCoA:ICA)	8:1:14:1:3	
Operation type (clipping:coiling:none)	14:8:5	
Pure SAH:SAH + ICH	18:9	
Hemisphere ratio (Rt:Lt)	13:14	

Values represent mean (± standard deviation).

SAH: subarachnoid hemorrhage; ACoA: anterior communicating; ACA: anterior cerebral artery; MCA: middle cerebral artery; PCoA: posterior communicating artery; ICA: internal carotid artery; ICH: intracerebral hemorrhage.

### Diffusion tensor imaging

**Figure 1:**
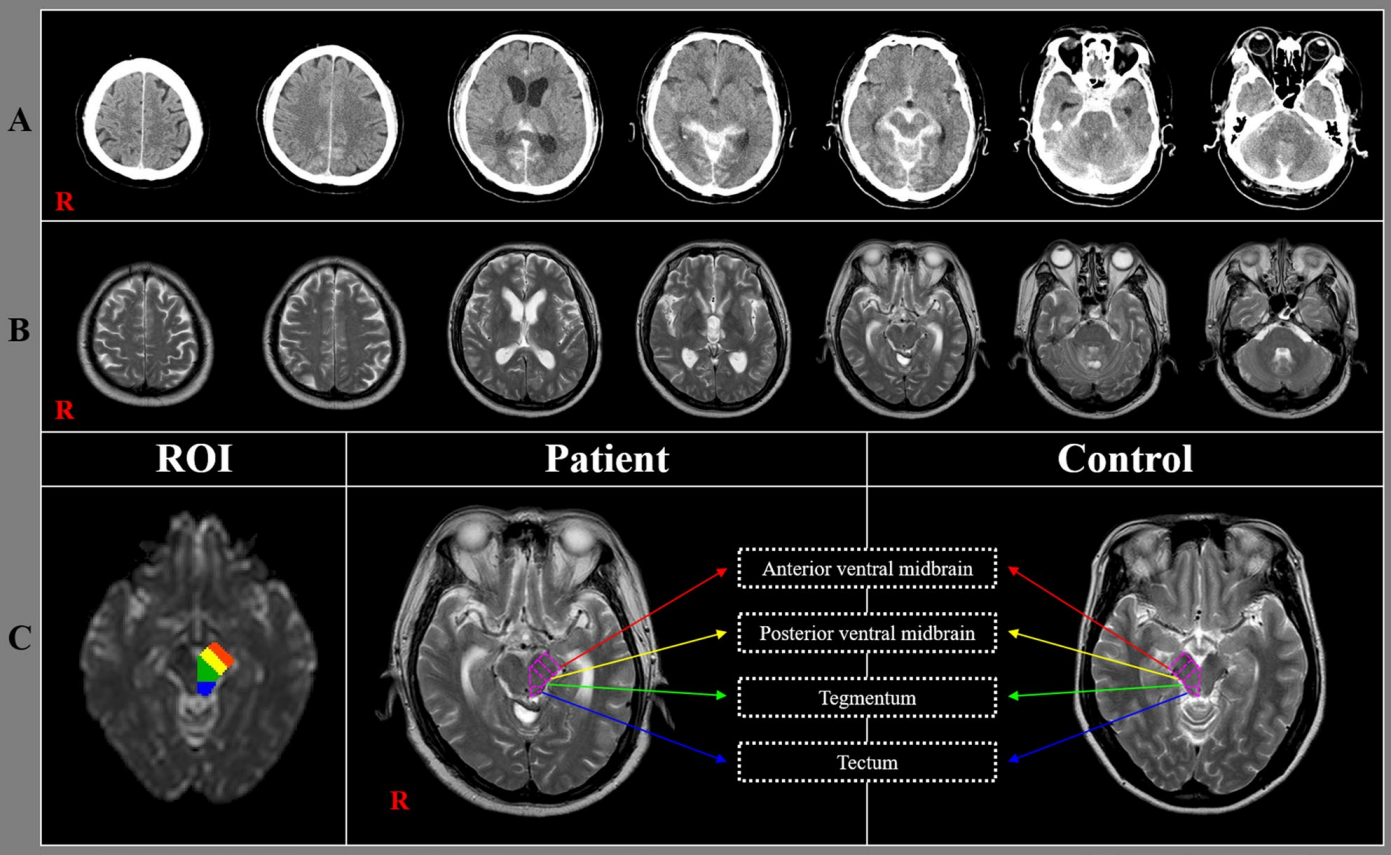
(**A**) Brain computed tomography images at onset reveal subarachnoid hemorrhage in a representative subject (68-year-old male) of the patient group. (**B**) T2-weighted brain magnetic resonance images obtained at the time of diffusion tensor imaging scanning; (**C**) Four regions of the midbrain examined by diffusion tensor imaging of the midbrain.

The DTI scans were obtained at an average of 2.18 ± 2.12 months after SAH onset. DTI data were acquired by using a six-channel head coil on a 1.5 T Philips Gyroscan Intera (Philips, Best, Netherlands). For each of the 32 non-collinear diffusion-sensitizing gradients, 70 contiguous slices were acquired parallel to the anterior commissure–posterior commissure line. Imaging parameters were as follows: acquisition matrix = 96 × 96; reconstructed matrix = 192 × 192; field of view = 240 mm × 240 mm; TR = 10.726 ms; TE = 76 ms; parallel imaging reduction factor (SENSE factor) = 2; EPI factor = 49; b = 1000 s/mm^2^; NEX = 1; and slice thickness = 2.5 mm with no gap. For analysis of the DTI data for the midbrain, the Oxford Centre for Functional Magnetic Resonance Imaging of the Brain (FMRIB) Software Library (http://www.fmrib.ox.ac.uk/fsl) was used. Affine multi-scale two-dimensional registration was used for correction of head motion effects and image distortion due to eddy currents. Fiber tracking was performed by using a probabilistic tractography method based on a multi-fiber model and was applied in the current study by utilizing tractography routines implemented in FMRIB Diffusion software (5000 streamline samples, 0.5 mm step lengths, curvature threshold = 0.2). We chose the midbrain in 27 hemispheres that did not show any pathology other than SAH and 25 hemispheres in 25 control subjects. The regions of interest were placed on four regions of the midbrain (the anterior portion of ventral midbrain, the posterior portion of the ventral midbrain, the tegmentum [posterior to the substantia nigra and ventral to the cerebral aqueduct], and tectum [posterior area of the cerebral aqueduct])^27,28^ (Fig. 1). In this study, the ventral midbrain was comprised of the cerebral peduncles, substantia nigra, crus cerebri, and corticobulbar fibers^27^. Fractional anisotropy (FA) and mean diffusivity (MD) values of the four midbrain regions were obtained for each hemisphere.

### Statistical analysis

Statistical analysis was performed by using SPSS 21.0 for Windows (SPSS, Chicago, IL, USA). The chi-squared test was used to determine the significance of differences in sex composition and hemisphere ratio of the patient and control groups. An independent *t*-test was performed to compare mean FA values between the patient and control groups. One-way analysis of variance (ANOVA) and least significant difference (LSD) post hoc testing was performed to determine the significance of differences among the FA and MD values of the anterior ventral midbrain, posterior ventral midbrain, tegmentum, and tectum regions in the patient and control groups. Analysis of covariance (ANCOVA) was used to determine the association between possible confounding factors (age, sex and hemisphere ratio) that could affect FA and MD values of the anterior ventral midbrain, posterior ventral midbrain, tegmentum, and tectum regions in the patient groups. The confounding factors (age, sex and hemisphere ratio) were used as a covariate via ANCOVA.

### Ethical approval

All procedures performed in the studies involving human participants were in accordance with the ethical standards of the institutional and/or national research committee and with the 1964 Helsinki Declaration and its later amendments or comparable ethical standards. For this type of study formal consent is not required.

### Informed consent

Informed consent was obtained from all individual participants included in the study.

## Results

Comparisons of the FA and MD values of the anterior ventral midbrain, posterior ventral midbrain, tegmentum, and tectum regions of the midbrain between the patient and control subjects are summarized in Tables 2 and 3. The mean FA values of each of the four regions of the midbrain (anterior ventral midbrain, posterior ventral midbrain, tegmentum, and tectum) of the patient group were significantly lower than those of the control group (*p* < 0.05). In the patient group, the mean FA values of the anterior ventral midbrain, posterior ventral midbrain, tegmentum, and tectum were significantly different (ANOVA; F = 3.22, *p* < 0.05). Post hoc LSD test results were observed that the mean FA value of the anterior ventral midbrain was significantly lower than those of the posterior ventral midbrain, tegmentum, and tectum (*p* < 0.05); in contrast, there were no differences in mean FA values of the posterior ventral midbrain, tegmentum, and tectum (*p* > 0.05). The mean MD values of each of the four regions of the midbrain (anterior ventral midbrain, posterior ventral midbrain, tegmentum and tectum) of the patient group were significantly higher than those of the control group (*p* < 0.05). In the patient group, the mean MD values of the anterior ventral midbrain, posterior ventral midbrain, tegmentum, and tectum were not significantly different (ANOVA; F = 0.77, *p* > 0.05). In the ANCOVA, although the age, sex and hemisphere ratio significantly affected the mean FA values of the anterior ventral midbrain, posterior ventral midbrain, tegmentum, and tectum (F = 4.27, 7.51, 0.04, respectively, p < 0.05), the mean FA values of the anterior ventral midbrain, posterior ventral midbrain, tegmentum, and tectum were significantly different (F = 5.92, p < 0.05). Regarding the mean MD value, the age and sex significantly affected the mean MD values of the anterior ventral midbrain, posterior ventral midbrain, tegmentum, and tectum (F = 39.97, 7.90, p < 0.05), whereas the hemisphere ratio did not significantly affect (F = 1.21, p > 0.05). By contrast, the mean MD values of the anterior ventral midbrain, posterior ventral midbrain, tegmentum, and tectum were not significantly different (F = 1.08, p > 0.05).

**Table 2:** Comparison of mean fractional anisotropy and mean diffusivity levels of four midbrain regions between the patient and control groups.

		Anterior ventral midbrain	Posterior ventral midbrain	Tegmentum	Tectum
FA	Patient group	0.327 (± 0.02)	0.340 (± 0.02)	0.343 (± 0.02)	0.343 (± 0.02)
	Control group	0.347 (± 0.02)	0.354 (± 0.02)	0.355 (± 0.02)	0.363 (± 0.02)
*p*		< 0.00*	0.01*	0.03*	< 0.00*
MD	Patient group	0.983 (± 0.05)	0.982 (± 0.05)	0.974 (± 0.06)	0.996 (± 0.06)
	Control group	0.930 (± 0.04)	0.930 (± 0.04)	0.936 (± 0.04)	0.961 (± 0.06)
*p*		< 0.00*	< 0.00*	0.01*	0.04*

Values represent mean (± standard deviation).

FA: fractional anisotropy; MD: mean diffusivity.

*Significant difference between the patient and control groups, *p* < 0.05.

**Table 3:** Comparison of mean fractional anisotropy levels of four midbrain regions in the patient group.

		Anterior ventral midbrain	Posterior ventral midbrain	Tegmentum	tectum	F	p
ANOVA	FA	0.327 (± 0.02)^a^	0.340 (± 0.02)^b^	0.343 (± 0.02)^b^	0.343 (± 0.02)^b^	3.22	0.03*
	MD	0.982 (± 0.05)	0.982 (± 0.04)	0.974 (± 0.06)	0.996 (± 0.06)	0.77	0.51
ANCOVA	FA	F (patient group, age, sex, hemisphere ratio)			5.92, 4.27, 7.51, 0.04		
		*P* (patient group, age, sex, hemisphere ratio)			< 0.00*, < 0.00*, 0.01*, 0.04*		
	MD	F (patient group, age, sex, hemisphere ratio)			1.08, 39.97, 7.90, 1.21		
		*P* (patient group, age, sex, hemisphere ratio)			*0.36,* < 0.00*, 0.01*, 0.27		

Values represent mean (± standard deviation); FA: fractional anisotropy; MD: mean diffusivity; One-way ANOVA and the LSD post hoc test were used for comparison of diffusion tensor parameter; ANCOVA were used to determine the association between possible confounding factors (age, sex and hemisphere ratio and diffusion tensor parameter.

Significant values are italics.

*Significant difference between the patient and control groups at the indicated *p.*

LSD test result: a < b.

## Discussion

In the current study, we used DTI to assess midbrain injuries in patients with SAH and obtained the following results: (1) the mean FA and MD values of each of the four regions of the midbrain in the patient group were significantly lower and higher than those of the control group, respectively, (2) in the patient group, the mean FA value of the anterior ventral midbrain was lower than those of the posterior ventral midbrain, tegmentum, and tectum. However, no significant difference was observed among the mean FA values of the posterior ventral midbrain, tegmentum, and tectum. However, differences were not observed among four regions of the midbrain (anterior ventral midbrain, posterior ventral midbrain, tegmentum and tectum) in the mean MD values, (3) the mean FA values of the anterior ventral midbrain, posterior ventral midbrain, tegmentum and tectum were significantly affected by the age, sex and hemisphere ratio. In addition, the mean MD values of the anterior ventral midbrain, posterior ventral midbrain, tegmentum and tectum were also significantly affected by the age and sex except for the hemisphere ratio.

Among the various DTI parameters, the FA and MD is the most commonly used diagnostic parameter^21,22,24^. The FA value indicates the degree of directionality of water diffusion such as that associated with axons, myelin, or microtubules and has a range of zero (completely isotropic diffusion) to one (completely anisotropic diffusion)^21,22^. Therefore, a reduced FA value is indicative of injury to the neural structure^21,22^. The MD value indicates the magnitude of water diffusion in tissue which can increase with some forms of pathology or neuronal injury^24^. Consequently, our results showing that the FA and MD values of each of the four regions of the midbrain in the patient group were significantly lower (FA) and higher (MD) than those of the control group indicate the presence of injury in each of the four midbrain regions in the patient group. In addition, the mean FA value of the anterior ventral midbrain was lower than those of the posterior ventral midbrain, tegmentum, and tectum, which were not significantly different. Thus, our results suggest that all four regions of the midbrain in the patient group were injured with the anterior ventral midbrain injury being the most severely injured among the four midbrain regions.

The results showing injuries in all four regions of the midbrain suggest that all regions of the midbrain may be injured irrespective of the area that is interfaced with the subarachnoid space by the SAH. This is consistent with results presented in previous studies which demonstrated that neural structures distant from the subarachnoid space may be injured following a SAH^6,9,12,14^. The results showing that the anterior ventral midbrain injury was the most severely injured among the four midbrain regions may be attributed to that area having the largest surface area interfacing with the subarachnoid space.

The precise pathophysiological mechanisms associated with SAH-related brain injury have not been clearly elucidated. However, several mechanisms have been suggested: global vasogenic edema in both white and deep gray matter, vasospasm and cerebral ischemia, mechanical injury (via increased intracranial pressure by or direct mass effect of the SAH), and chemical injury (a blood clot can cause neural injury by release of potentially damaging substances, such as free iron, which may result in the generation of free radicals or inflammatory cytokines)^5,7,25,29–33^. Among the above pathophysiological mechanisms, our results suggest that SAH- produced mechanical or chemical injury may be the two most plausible pathophysiological mechanisms. The results of the present study are consistent with those in previous studies that have reported the injury of neural structures adjacent to the subarachnoid space following SAH^7,8,10,11,13,15^.

Following the introduction of DTI, a few studies have reported on injuries of neural tracts that pass through the midbrain^7,9,10^. In 2012, Yeo et al. reported that the FA value of the corticospinal tract (CST) area of the midbrain was significantly decreased, but without significant changes in other regions, in 22 patients with SAH by using DTI^7^. Consequently, the authors suggested that the CST could be vulnerable to SAH in the midbrain area because SAH frequently occurs in a perimesencephalic cistern, and the location of the CST is close to this cistern in the midbrain^25,34^. Subsequently, Jang et al.^9^, by using DTI, observed that 52.9% of 34 hemispheres showed a discontinuation of the corticoreticular pathway at the midbrain level in 17 patients with SAH^9^. Furthermore, the FA value of the corticoreticular pathway showed a moderate positive correlation with the subjects’ motor weakness of proximal joint muscles (shoulder and hip). As a result, the authors concluded that injury of the corticoreticular pathway may be common in patients with motor weakness of the proximal joint muscles after SAH. During the same year, Jang and Kim^10^ used DTI to demonstrate injury of the ascending reticular activating system (ARAS) between the pontine reticular formation and the intralaminar thalamic nuclei based on the Glasgow Coma Scale scores of the patients with impaired consciousness, which showed a strong positive correlation with the ARAS fiber numbers in 24 patients with SAH^10^. Consequently, the authors assumed that ARAS injury might be associated with SAH in the subarachnoid space around the thalamus and brain stem.

Irrespective of the findings in those studies, to the best of our knowledge, this is the first DTI-based study on midbrain injuries in patients with SAH. However, several limitations of this study should be considered. First, DTI analysis is operator dependent and has inability to calculate the heterogeneity of fiber structures within a voxel^35,36^. Second, we were unable to obtain clinical data for the subjects because this study was performed retrospectively. Third, the study included a relatively small number of patients. Fourth, we could not control the sample size and location of the aneurysms, and correlate with clinical outcomes because this study was a retrospective study. Therefore, further prospective studies that can include clinical data and a larger number of patients should be encouraged.

In conclusion, we detected evidence of neural injury in all four regions of the midbrains of patients with SAH, and the results showed that the anterior ventral midbrain was the most severely injured among the four midbrain regions. Our results suggest that the pathophysiological mechanism of these neural injuries might be related to the occurrence of a subarachnoid hematoma.
